# Prioritizing drug targets in systemic lupus erythematosus from a genetic perspective: a druggable genome-wide Mendelian randomization study

**DOI:** 10.1007/s10067-024-07059-3

**Published:** 2024-07-13

**Authors:** Yuan Gao, Youtao Zhou, Zikai Lin, Fengzhen Chen, Haiyang Wu, Chusheng Peng, Yingying Xie

**Affiliations:** 1 The First Clinical College, Guangzhou Medical University, Guangzhou, China; 2 Nanshan College, Guangzhou Medical University, Guangzhou, China; 3 The Second Clinical College, Guangzhou Medical University, Guangzhou, China; 4 Department of Rheumatology and Immunology, The First Affiliated Hospital of Guangzhou Medical University, Guangzhou Medical University, No. 151, Yanjiang West Road, Yuexiu District, Guangzhou, China

**Keywords:** Drug target, Expression quantitative trait loci, Genetics, Mendelian randomization, Systemic lupus erythematosus

## Abstract

**Objectives:**

Systemic lupus erythematosus (SLE) is a heterogeneous autoimmune disease with an unsatisfactory state of treatment. We aim to explore novel targets for SLE from a genetic standpoint.

**Methods:**

Cis-expression quantitative trait loci (eQTLs) for whole blood from 31,684 samples provided by the eQTLGen Consortium as well as two large SLE cohorts were utilized for screening and validating genes causally associated with SLE. Colocalization analysis was employed to further investigate whether changes in the expression of risk genes, as indicated by GWAS signals, influence the occurrence and development of SLE. Targets identified for drug development were evaluated for potential side effects using a phenome-wide association study (PheWAS). Based on the multiple databases, we explored the interactions between drugs and genes for drug prediction and the assessment of current medications.

**Results:**

The analysis comprised 5427 druggable genes in total. The two-sample Mendelian randomization (MR) in the discovery phase identified 20 genes causally associated with SLE and validated 8 genes in the replication phase. Colocalization analysis ultimately identified five genes (*BLK*, *HIST1H3H*, *HSPA1A*, *IL12A*, *NEU1*) with PPH4 > 0.8. PheWAS further indicated that drugs acting on *BLK* and *IL12A* are less likely to have potential side effects, while *HSPA1A* and *NEU1* were associated with other traits. Four genes (*BLK*, *HSPA1A*, *IL12A*, *NEU1*) have been targeted for drug development in autoimmune diseases and other conditions.

**Conclusions:**

.This study identified five genes as therapeutic targets for SLE. Repurposing and developing drugs targeting these genes is anticipated to improve the existing treatment state for SLE.

**Table:** 

**Key Points**
• *We identified five gene targets of priority for the treatment of SLE, with BLK and IL12A indicating fewer side effects.* • *Among the existing drugs that target these candidate genes, Ustekinumab, Ebdarokimab, and Briakinumab (targeting the IL12 gene) and CD24FC (targeting HSPA1A) may potentially be repurposed for the treatment of SLE.*

**Supplementary Information:**

The online version contains supplementary material available at 10.1007/s10067-024-07059-3.

## Introduction

Systemic lupus erythematosus (SLE) is a typical systemic autoimmune illness that is marked by aberrant immune system activity, leading to a variety of symptoms such as rashes, joint pain, kidney, and nervous system damage [1]. The estimated overall incidence rate of SLE in the United States is 5.1 per 10,000 individuals, with a prevalence rate of around 72.8 per 10,000 individuals [2]. SLE predominantly affects young women with a long course and causes great suffering. Moreover, it poses a threat to general public health and has a major effect on patients’ physical functions, psychological and emotional states, as well as their social life [3]. SLE represents a serious societal and public health issue. Due to its chronic, recurrent nature, and the involvement of multiple organs, SLE patients require a substantial amount of healthcare resources and incur significant medical expenses [4].

Even as a common disease, the treatment of SLE remains challenging. First, due to genetic and phenotypic heterogeneity, SLE lacks specific therapeutic approaches. Corticosteroids and immunosuppressants are classic treatment methods in clinical practice, but many people still fail to respond to treatments [5]. And current management is unable to achieve a cure, with persistent disease activity often occurring after treatment [6]. Second, the complex immune dysregulation leads to significant individual variations in treatment responses. Most patients experience a chronic cycle of remission and relapse, with only a minority achieving long-term remission [7]. Third, in addition to the beneficial effects of treatment, prolonged use of corticosteroids and immunosuppressants can cause serious adverse reactions and significant toxicity in patients, including infections, organ damage, and even death [8]. Therefore, alongside refining management strategies, effective intervention and treatment for SLE are urgently needed.

For decades, efforts have been dedicated to exploring new therapeutic drugs. Randomized controlled trials (RCTs) are acknowledged as authoritative for researching drug actions and development. Unfortunately, these scientific experiments have not translated into clinically satisfactory treatment strategies [9]. The high heterogeneity, complex pathophysiology, and lack of effective biomarkers are the main reasons for this stagnation [10]. Linking human genetics with medication research could help accelerate this procedure, and selecting targets supported by genetic associations can significantly increase the probability of medication approval via clinical trials [11]. The advent and rapid development of genome-wide association studies (GWASs) have contributed to recognizing the relationship between genetic variations and SLE. Although GWAS has discovered numerous genetic loci linked to the risk of SLE [12], this approach cannot reliably confirm causality or effectively promote medication development.

Mendelian randomization (MR) is frequently applied to evaluate the causal relationships between variable exposures and outcomes as a genetic tool for instrumental variable analysis [13]. MR analysis is based on the natural random allocation of genetic variants during meiosis and is referred to as “nature’s randomized double-blind controlled trial [14].” Genes implicated in drug actions are considered exposures, with individuals receiving different levels of gene expression due to random allocation. Expression quantitative trait loci (eQTLs) are genetic variants correlated with gene expression that are retrieved as instrumental variables (IVs). By conducting MR on eQTLs and disease GWAS, the causal link between gene expression levels and a certain disease risk is explored, thereby identifying drug treatment targets for the disease, which guides drug development and repurposing [15]. Currently, druggable genome-wide MR analysis, integrating eQTLs and disease GWAS summary data, has been utilized to identify therapeutic targets in various immune-related diseases [16, 17]. However, to date, there has been no research integrating GWAS, gene expression eQTLs, and pharmacogenomic data to explore drug targets for SLE.

In brief, we aim to confirm novel pharmacological targets for SLE. Our study represents the first druggable genome-wide MR research in the field of SLE, identifying new targets for drug treatment, which may play a significant role in drug clinical trials.

## Materials and methods

**Fig. 1:**
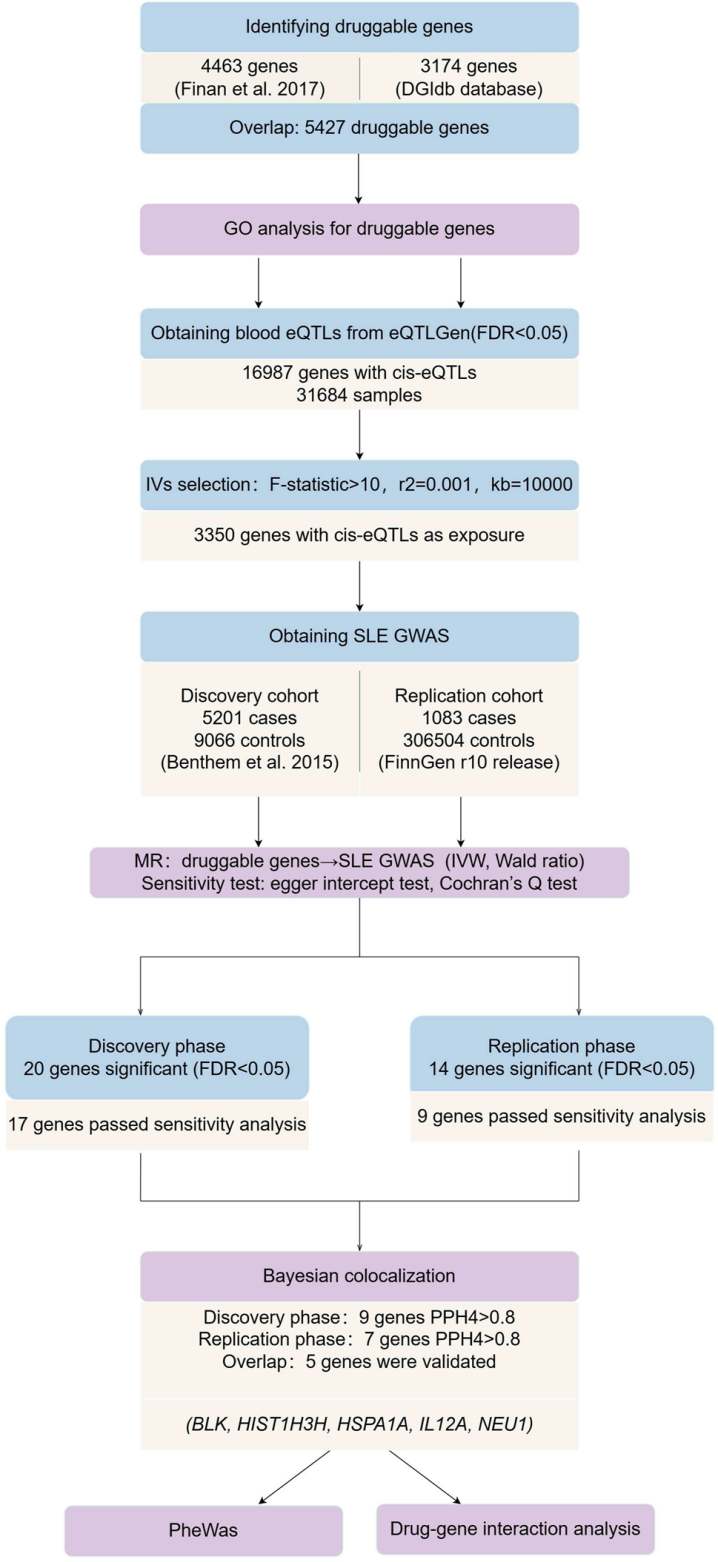
The overview of the study design. First, we acquired druggable genes. Second, GO analysis was conducted. Third, we performed two-sample MR in the discovery and replication phases using blood cis-eQTLs and two large SLE cohorts, respectively. Then, colocalization analyses were then conducted to clarify the robustness of the results. Moreover, we assessed the side effects of drugs that target the confirmed genes through PheWAS. Finally, we made drug predictions of targets and analyzed the pharmacogenomic interactions of existing drugs via multiple databases. GO, gene ontology; MR, Mendelian randomization; eQTL, expression quantitative trait loci; SLE, systemic lupus erythematosus; PheWAS, phenome-wide association analysis

This study primarily focuses on Europeans. The overall research design is displayed in Fig. 1. First, we identified 5427 druggable genes. Second, GO analysis was conducted. Third, we extracted cis-eQTLs for these genes to gain insight into the causal effects of druggable gene expression on SLE. Then, colocalization analysis was performed to explore whether gene expression and SLE risk are driven by the same genetic variations. The robustness of the results was verified through replication with different SLE cohorts. Moreover, we carried out a PheWAS analysis to find out how candidate genes relate to other phenotypes, aiming to assess the pleiotropy of genes and potential side effects of drugs. Finally, we made drug predictions of candidate targets and analyzed the pharmacogenomic interactions of existing drugs. More details about the methods are described below.

### Identifying genes susceptible to pharmacological intervention

We obtained druggable genes from the study by Finan et al. [18] and the Drug-Gene Interaction Database (DGIdb v4.2.0, www.dgidb.org). The Finan team linked genes, drugs, and clinical indications, leveraging studies on targetable genes for selecting and confirming drug targets in human diseases. Based on this, we extracted 4463 genes (Online Resource: Table S1). Information on drug-gene interactions and druggable genes is available from multiple sources in the DGIdb database. We downloaded the “interactions” data updated in February 2022 from this database and extracted information on 3174 genes (Online Resource: Table S2). By intersecting the genes from the two datasets, we identified 5427 actionable druggable genes for further analysis (Online Resource: Table S3).

### GO analysis for potentially druggable genes

Gene Ontology (GO) analysis was conducted to initially investigate the biological functions and potential biological effects of the 5427 candidate genes in SLE. The database for annotation, visualization, and integrated discovery (DAVID, https://david.ncifcrf.gov/), a comprehensive bioinformatics analysis tool, was used for this analysis. Results with FDR < 0.05 were considered significant, and gene ratio was defined as the ratio of enriched genes in a pathway to the total number of genes involved in the GO analysis.

### Acquisition of blood cis-eQTLs

Blood cis-eQTL data were sourced from eQTLGen (https://eqtlgen.org/). The data resulted from a large-scale meta-analysis of whole blood samples from 31,684 healthy individuals of European ancestry, drawn from 37 cohorts, including cis-eQTL data for 16,987 identifiable genes. A detailed description of the data could be found in this original article [19]. We downloaded significant cis-eQTLs (false discovery rate [FDR] < 0.05) and allele frequency information. SNPs located within 1 Mb of the gene center and tested in at least two cohorts were included as eQTLs. Information on data details can be found in Online Resource: Table S4.

### Acquisition of SLE GWAS

We selected the GWAS summary dataset from a large-scale SLE study as our discovery cohort [20]. The study included 14,267 participants of European ancestry, comprising 5201 SLE cases and 9066 healthy controls. The diagnosis of SLE conformed to the standards of the American College of Rheumatology (ACR). The raw data is downloaded from the IEU OpenGWAS website (https://gwas.mrcieu.ac.uk/, ID: ebi-a-GCST003156). For the replication phase, we utilized the latest SLE GWAS data (ID: finngen_R10_SLE_FG) released in December 2023 from the FinnGen database (https://r10.finngen.fi/), which includes 1083 cases and 306,504 healthy controls from the European population. The baseline characteristics are presented in Online Resource: Table S5. Both exposure and outcome samples were predominantly from European populations, avoiding population heterogeneity. And the samples were from different studies with no population overlap.

### eQTL MR analysis

Version 0.5.6 of the TwoSampleMR package was used to perform MR analysis. The selection of genetic IVs was based on three assumptions [13, 21]: (1) strong association with the exposure; (2) not associated with confounding factors; and (3) association with the outcome only through the exposure. To minimize the bias introduced by low-quality IVs, we calculated the F-statistic, and those with a value less than 10 were excluded. To reduce confounding and pleiotropy, SNPs with *r*^2^ > 0.001 for the most significant SNPs were removed within 10,000 kb using European samples from the 1000 Genomes Project [22], leaving SNPs with no linkage disequilibrium as IVs. Data on exposure and results were combined and further loaded. Within the primary MR analysis, when nSNP = 1, the Wald ratio was used to calculate its MR estimate. When nSNP ≥ 2, the inverse variance weighted (IVW), MR-Egger, and weighted median methods were utilized. Odds ratios (OR) and 95% confidence intervals (CI) were used to estimate the relationship between gene expression and SLE risk, with OR > 1 indicating that increased gene expression was associated with an increased risk of SLE. For genes containing more than two genetic variants, we applied the MR Egger intercept test to assess the level of deviation of the intercept from zero to account for potential pleiotropy between exposure and outcome. A *p* value < 0.05 is considered significant. Furthermore, we performed Cochran’s Q test to assess heterogeneity. To avoid false positives caused by multiple testing, corrections for multiple hypothesis testing are executed. In the discovery phase, we calculated FDR, and FDR < 0.05 was defined as significant. During the replication stage, a *p* value less than 0.05 was deemed noteworthy.

### Colocalization analysis

Focusing on genes within the significance threshold in both the discovery and replication phases of MR analysis, we continued the following colocalization analysis to test whether the two phenotypes (gene expression and SLE risk) share the same causal variant in the given region, using the coloc package. Prior probabilities are set to default values: P1 denotes the probability that an SNP is significantly associated with gene expression (P1 = 1 × 10^–4^), P2 indicates the probability that an SNP deals with SLE risk (P2 = 1 × 10^–4^), and P12 represents the probability that an SNP is related to both traits (P12 = 1 × 10^–5^). The posterior probabilities conform to one of the five hypotheses [23]. This study aims to test hypothesis 4 (PPH4), and the threshold of colocalization analysis is set at PPH4 > 0.8.

### Phenome-wide association analysis

We conducted a phenome-wide scan to identify associations between confirmed drug targets and the entire phenome, thereby exploring the potential pleiotropy and side effects of targeted drugs to better guide drug development. The entire phenome data were derived from UK Biobank participants of British white descent, with all individuals assessed using TOPMed imputation and binary outcomes analyzed using SAIGE. We downloaded the UK Biobank TOPMed dataset from the PheWeb website version 1.3.15 (https://pheweb.org/UKB-TOPMed/), which includes 1,419 phenotypes. Using the genes identified above as exposures, a phenome-wide MR analysis was carried out with the corresponding eQTLs and outcome GWAS, maintaining the same parameter settings as previously described. FDR = 0.05 was established for the significance threshold.

### Drug prediction and evaluation of existing drugs

To assess the druggability of genes, we utilized the Enrichr tool (https://maayanlab.cloud/Enrichr/) to analyze the interactions between target genes and linked drugs or compounds. The identified genes were uploaded, and the DSigDB database, focusing on disease or drug characteristics, was selected for analysis. Furthermore, we also searched the DrugBank database (https://go.drugbank.com/) and the ChEMBL database (www.ebi.ac.uk/chembl/) to explore existing drugs targeting the identified genes and to assess the capacity for drug repurposing.

### Statistics

Statistical calculations were conducted with the R software, version 4.2.2., and all genomic data were analyzed using the hg19 version (GRCH37) except for SLE cohort data from FinnGen. The data used in the study are publicly available, so ethical approval was not required.

## Results

### Annotations and enrichment results of GO analysis

**Fig. 2:**
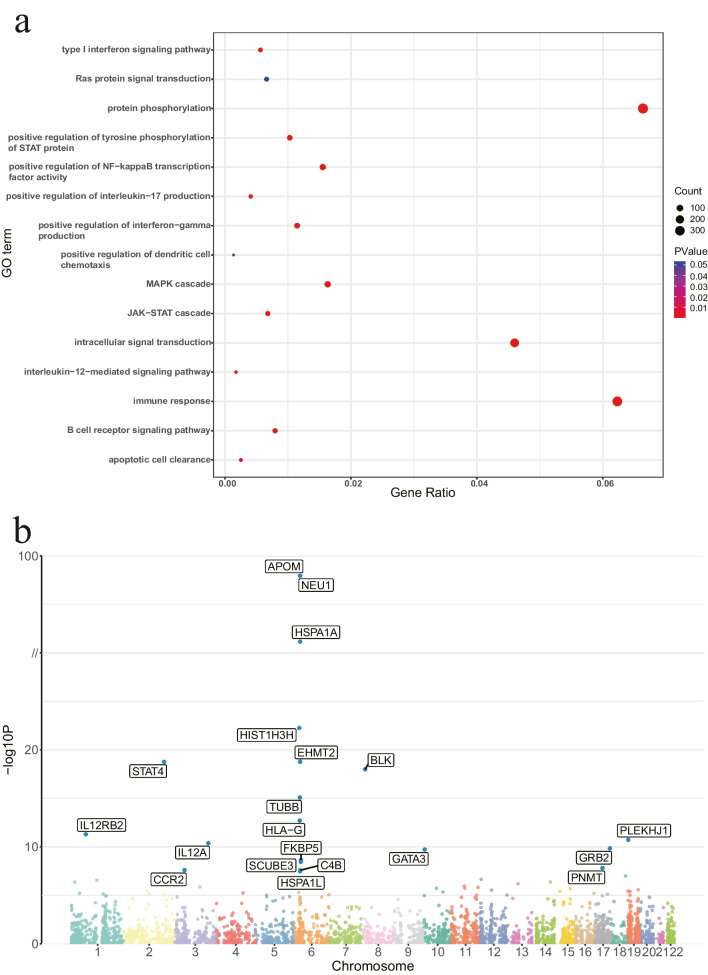
Enrichment bubble map for GO analysis of genes and Manhattan plot for MR analysis in the discovery phase. **a** Bubble map of 15 biological processes (BP) obtained by GO analysis of candidate genes. Abscissa represents the gene ratio and the ordinate represents the go entry name. Bubble size represents the count of genes and the color represents the corrected *P* value. **b** MR results in the discovery phase revealed that 20 genes are significantly causally associated with SLE (FDR < 0.05). These genes are labeled and highlighted with blue dots in the plot. MR, Mendelian randomization; SLE, systemic lupus erythematosus; FDR: false discovery rate

GO analysis identified the relationships between genes and GO terms at the cellular component (CC), biological process (BP), and molecular function (MF) levels, with candidate genes significantly enriched in a total of 1297 BPs (FDR < 0.05) (Online Resource: Table S6). Consistent with the pathophysiology of SLE, genes are mainly involved in BPs like signal transduction, inflammatory responses, and immunological activities. We displayed 15 pathways of interest with genes significantly enriched, such as immune response (GO:0006955), type I interferon signaling pathway (GO:0060337), MAPK cascade (GO:0000165), and JAK-STAT cascade (GO:0007259) (Fig. 2a).

### Acquisition of druggable genes and blood cis-eQTLs

The original significant blood cis-eQTL data (FDR < 0.05) includes 16,987 genes and 10,507,664 SNPs. After intersecting with the previously obtained 5427 druggable genes, calculating the F-statistic, and removing exposures with F < 10, we obtained 3404 genes. After the SNPs were clumped, 3350 genes remained for subsequent MR analysis.

### Twenty genes with a significant causal association with SLE risk were identified in the discovery phase

**Fig. 3:**
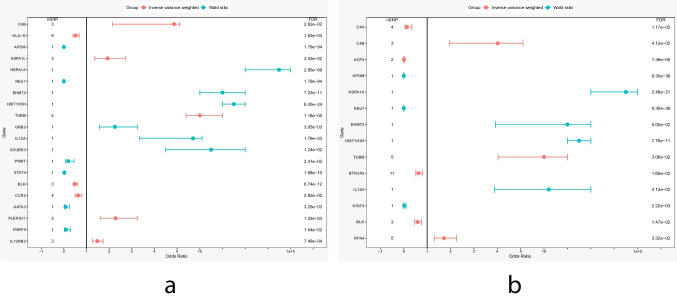
Forest plots for MR results of genes causally associated with SLE in the discovery and replication phases. **a** Forest plot for MR results of 20 significant genes identified in the discovery phase. **b** Forest plot for MR results of 14 significant genes identified in the replication phase. The threshold of significance was set at FDR < 0.05. The red line represents the estimates using the IVW method, and the blue line represents the estimates using the Wald ratio method. MR, Mendelian randomization; SLE, systemic lupus erythematosus; FDR: false discovery rate

The cohort for the discovery phase comes from the study by Bentham et al.[20], including 9066 controls and 5201 SLE cases of European ancestry. MR was performed and FDR correction was applied to the results of the IVW or Wald ratio, resulting in the discovery of 20 genes whose expression is causally correlated with the risk of SLE (FDR < 0.05) (Figs. 2b and 3a and Online Resource: Table S7). Seventeen genes passed the sensitivity analysis, but the heterogeneity test was not passed by *C4B*, *HLA-G*, and *TUBB* (Q_pval < 0.05), and they were therefore excluded from further analysis.

### Fourteen genes with a significant causal association with SLE risk were identified in the replication phase

The SLE cohort for the replication phase comes from the FinnGen consortium, comprising 1083 cases and 306,504 controls of European ancestry. Exposure data utilized the same blood eQTLs as in the discovery phase and conducted MR analysis following the same process and parameters. Using the IVW or Wald ratio method, 14 genes were found to be causally linked with SLE risk (*p* < 0.05) (Fig. 3b and Online Resource: Table S8). Nine genes passed sensitivity analysis, of which 8 genes at the discovery stage were validated (Online Resource: Table S9). The results of the discovery and replication phases suggested that the expression of *BLK* and *NEU1* were protective factors for SLE (OR < 1), whereas the expressions of *HIST1H3H*, *HSPA1A*, and *IL12A* were risk factors for SLE (OR > 1).

### Five genes were prioritized in colocalization analysis

Studies have shown that genes that are analyzed by both MR and colocalization have a higher chance of being confirmed as drug targets [24]. This study aims to test hypothesis 4 of colocalization, conducting colocalization analyses separately for genes identified in both the discovery and replication phases. Most of the genes produced significant results (PPH4 > 0.8), with 9 out of 17 genes in the discovery phase and 7 out of 9 genes in the replication phase reaching the analysis threshold (Online Resource: Tables S10–S11). Among the 8 genes validated in the replication phase, five genes (*BLK*, *HIST1H3H*, *HSPA1A*, *IL12A*, *NEU1*) reached significant levels in the colocalization analysis across both phases (Table 1). Therefore, we speculate that the expression of these 5 genes and the risk of SLE are each driven by a causal site.

**Table 1:** Colocalization results of 8 genes verified in the replication phase

Genes	PPH0	PPH1	PPH2	PPH3	PPH4
BLK	0.000	0.000	0.000	0.037	0.963
HIST1H3H	0.000	0.002	0.000	0.029	0.969
HSPA1A	0.000	0.002	0.000	0.002	0.996
IL12A	0.000	0.000	0.000	0.045	0.955
NEU1	0.000	0.000	0.000	0.101	0.899
APOM	0.000	0.000	0.000	0.320	0.680
EHMT2	0.000	0.000	0.000	1.000	0.000
STAT4	0.000	0.000	0.000	0.976	0.024

*PPH0-PPH4* represents the posterior probabilities conform to one of the five hypotheses. *PPH0* no association with either trait. *PPH1* association with trait 1, not with trait 2. *PPH2* association with trait 2, not with trait 1. *PPH3* association with trait 1 and trait 2, two independent SNPs. *PPH4* association with trait 1 and trait 2, one shared SNP

The significance threshold is set at PPH4 > 0.8

### PheWAS evaluated the side effects of drugs targeting the identified five genes

**Fig. 4:**
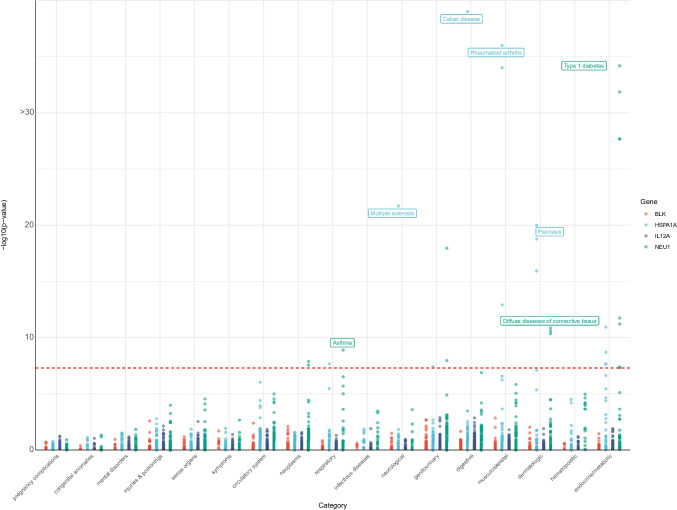
Manhattan plot for PheWAS of blood *BLK*, *HSPA1A*, *IL12A*, and *NEU1.* Each dot represents a disease trait within a specific category on the horizontal axis, with different colors representing three different genes. The red dashed line represents the significance threshold for the *p* value at 5e-08. Several phenotypes discussed in detail in this study are labeled

The phenome-wide scan investigated associations between potential targets and various traits, offering foresight into potential side effects in drug development. Through phenome-wide scanning, we found that two of the above five genes were associated with multiple phenotypes (p < 5E-08): *HSPA1A* is associated with several immune diseases, such as celiac disease, rheumatoid arthritis (RA), psoriasis, and multiple sclerosis (MS), suggesting that the different expression levels of *HSPA1A* may cause an imbalance of immune homeostasis and mediate a wide range of immune-related illnesses. *NEU1* is negatively associated with type 1 diabetes, diffuse diseases of connective tissue, and asthma, and its increased expression may reduce the risk of these diseases. However, no associations were found between *BLK* and *IL12A* and other traits, suggesting that drugs that target *BLK* and *IL12A* are less likely to have side effects. The SNPs corresponding to *HIST1H3H* were not found in the entire phenome data, so no results are presented. Results are shown in Fig. 4 and Online Resource: Tables S12–S15.

### Drug prediction and evaluation of existing drugs

According to the five target genes identified through MR and colocalization screening, we utilized the DSigDB database for predicting potentially useful medications. Applying adjusted *p* values, the top ten medications or compounds were selected (Online Resource: Table S16). AC1L1KON (AC1L1KON CTD 00000061) was identified as the most significant drug for *IL12A* and *HSPA1A*. Moreover, trichostatin (trichostatin A PC3 UP), a histone deacetylase inhibitor vorinostat (vorinostat PC3 UP), and a topoisomerase inhibitor irinotecan (irinotecan PC3 UP) were associated with the most genes. We also searched the DrugBank and ChEMBL databases and found that there are currently drugs available for several genes (Table 2). *HSPA1A* is linked to CD24FC, a medication used to treat severe acute respiratory syndrome and graft-versus-host disease (GVHD). *IL12A* is associated with Ustekinumab, Ebdarokimab, and Briakinumab, which are employed in the treatment or research of psoriasis and various autoimmune diseases. These drugs act as inhibitors of their targets, potentially reducing the risk of SLE. The expression of *BLK* and *NEU1* can reduce the risk of SLE. However, the related drugs for *BLK*, such as Fostamatinib and Zanubrutinib, and for *NEU1*, such as Oseltamivir, Acetylsalicylic acid, and Celecoxib, are inhibitors of their targets, which may potentially increase the risk of SLE. For genes without corresponding drugs, guiding clinical trials and developing new medications through genetics is of significant importance.

**Table 2:** Available drugs targeting five genes retrieved by ChEMBL and DrugBank

Genes	Effect	Protein	UniProt- ID	Drug/molecule	Actions	Phase	Indications	Source
BLK	Protective	Tyrosine-protein kinase Blk	P51451	Fostamatinib	Inhibitor	Approved, investigational	1) It is approved for the treatment of ITP in patients who have had insufficient response to previous therapy 2) It has been identified as a potential therapeutic for controlling ARDS in patients with severe COVID-19 3) It has been investigated for the treatment and basic science of RA	DrugBank
				Zanubrutinib	Inhibitor	Approved, investigational	1) It is indicated for the treatment of MCL, Waldenström’s macroglobulinemia, MZL, CLL or SLL, and refractory or relapsed follicular lymphoma 2) It is investigated for the treatment of lupus nephritis	DrugBank
HIST1H3H	Risk	H3 clustered histone 10	P68431	NA	NA	NA	NA	NA
HSPA1A	Risk	Heat shock 70 kDa protein 1A	P0DMV8	CD24FC (Efprezimod alfa)	NA	Phase 3	1) It is developed to address GVHD in stem cell transplantation in leukemia patients 2) It has been investigated for the treatment severe acute respiratory syndrome, neoplasms, and melanoma	CheMBL and DrugBank
IL12A	Risk	Interleukin-12 subunit alpha	P29459	Ustekinumab	Inhibitor	Approved	It is used to manage several inflammatory conditions such as plaque psoriasis, PsA, CD and IBD, and ulcerative colitis	CheMBL and DrugBank
				Ebdarokimab	Inhibitor	Phase 3	It is investigated for the treatment of psoriasis	
				Briakinumab	Inhibitor	Phase 3	It is investigated for use/treatment of several driven autoimmune diseases, such as psoriasis and psoriatic disorders, MS, CD, RA, and other autoimmune and inflammatory disorders	
NEU1	Protective	Neuraminidase 1/Sialidase-1	Q99519	Oseltamivir	Inhibitor	Approved	It is used in the prophylaxis and treatment of influenza	DrugBank
				Acetylsalicylic acid	Inhibitor	Approved	It is used to treat pain, fever, inflammation, migraines, and reducing the risk of major adverse cardiovascular events	DrugBank
				Celecoxib	Inhibitor	Approved, Investigational	1) It is used to treat OA, RA, acute pain, menstrual symptoms, and to reduce polyps is familial adenomatous polyposis 2) It is investigated for the treatment of AS and multiple cancers	DrugBank

*ITP* immune thrombocytopenic purpura, *ARDS* acute respiratory distress syndrome, *RA* rheumatoid arthritis, *MCL* mantle cell lymphoma, *MZL* marginal zone lymphoma, *CLL* chronic lymphocytic leukemia, *SLL* small lymphocytic lymphoma, *GVHD* graft-versus-host disease, *PsA* psoriatic arthritis, *CD* Crohn’s disease, *IBD* inflammatory bowel disease, *MS* multiple sclerosis, *OA* osteoarthritis, *AS* ankylosing spondylitis

## Discussion

As the first study in the field of SLE to integrate GWAS, gene expression eQTLs, and pharmacogenomic data to explore drug targets, we conducted a systematic analysis of the causal associations between 5427 druggable genes and SLE risk, ultimately identifying five potential therapeutic targets (*BLK*, *HIST1H3H*, *HSPA1A*, *IL12A*, *NEU1*). This work applied two-sample MR (IVW, MR-Egger and weighted median) to determine the causal link between gene expression and SLE risk, with sensitivity analyses utilized to exclude confounders such as horizontal pleiotropy and heterogeneity. Colocalization analysis was used to test whether gene expression and SLE risk share the same genetic variations. Among these genes, *IL12A* and *HSPA1A* are considered potential targets for existing drugs. PheWAS was also performed to explore the potential pleiotropy of target genes and the safety of corresponding drugs. A replication cohort was also set up to replicate the study, enhancing the reproducibility and credibility of the results.

*BLK* encodes a nonreceptor tyrosine kinase known for the Src oncogene family, engaged in cell proliferation and differentiation, primarily expressed in B cells, with roles in receptor signaling [25]. Geoffrey Hom’s group discovered *BLK* as an SLE susceptibility gene for the first time in 2008 [26]. The downregulation of this gene’s expression is linked to a higher chance of developing SLE and is considered to be involved in B-cell immune tolerance. Subsequent animal experiments also confirmed this finding, linking the reduction of *BLK* expression in mice to a dysregulated pro-inflammatory cytokine network and an increased risk of SLE [27]. Pharmacological studies have found that halofuginone can improve the disease progression in lupus mice. It is speculated that this therapeutic effect is achieved by promoting the mRNA expression of *BLK* or enhancing the kinase activity of *BLK*, thereby modulating immunity [28]. We found that higher *BLK* expression was connected with a lower risk of SLE (OR: 0.493), consistent with previous findings. PheWAS revealed that an increasing level of *BLK* expression is linked with a lower risk of RA, even though it did not reach our set significance threshold (beta = -0.316, *p* = 0.001). This suggests a trend indicating that the relationship between *BLK* and more autoimmune diseases requires further study in the future. Currently, two drugs (Fostamatinib and Zanubrutinib) targeting *BLK* have been approved for the market. Fostamatinib, acting on multiple targets and whose mechanism as an inhibitor of Syk has been confirmed, is now a first-line medication for Immune Thrombocytopenic Purpura (ITP). Syk signaling transduction is a component of immune cell autoreactive antibody activation, making Fostamatinib a candidate drug for autoimmune diseases [29]. The pathogenesis of SLE involves the generation and persistent existence of a significant amount of autoantibodies, and Syk inhibitors have been found to attenuate the inflammatory process in lupus models [30]. A phase II trial involving Fostamatinib in SLE patients was initiated but was discontinued due to commercial reasons (NCT00752999). Zanubrutinib is an inhibitor of both *BLK* and Bruton’s tyrosine kinase (*BTK*), targeting *BTK* for the treatment of multiple hematologic diseases such as mantle cell lymphoma (MCL). It is known that *BTK*, a TEC kinase family member, contributes to lupus autoimmunity as mice overexpressing *BTK* spontaneously develop lupus-like autoimmune pathology [31]. Zanubrutinib is currently undergoing a phase II trial with the purpose of treating active lupus nephritis (NCT04643470). Theoretically, the repurposing of Fostamatinib and Zanubrutinib for the treatment of SLE is feasible. However, as both drugs are also inhibitors of *BLK*, according to the findings of this study, inhibiting *BLK* may raise the chance of SLE. Therefore, additional research is required to weigh the relationships between multiple targets of drug action.

*IL12A* encodes the Interleukin-12 subunit alpha, a subunit of the cytokine that acts on T lymphocytes and natural killer cells. Together with IL12B, it forms IL12, playing a significant role in inflammatory responses and immune reactions [32]. *IL12A* positively promotes the expression of *IL12* [32]. Cellular experiments have indicated that Th1 cell development from CD4 + T cells is aided by IL12 [33]. Th1 cells are considered one of the pathogenic factors mediating autoimmune diseases, driving self-tissue damage through the secretion of IFN-γ [34]. SLE is an inflammatory disease linked to immune dysregulation, and the roles of Th1 cells and IL12 in the occurrence and development of SLE have been extensively studied [35]. A GWAS conducted in 2015 on samples of European ancestry mapped 10 new susceptibility loci, including *IL12A*, revealing *IL12A* as a risk factor for SLE (OR: 1.15), which was also validated in the GWAS among Asian populations [36]. Glucocorticoids are classical drugs in the clinical treatment of SLE, with one of their key actions being the reduction in the production of multiple pro-inflammatory cytokines, including IL12 [37]. A trial also demonstrated that corticosteroids significantly suppress *IL12* expression in the SLE treatment group, serving as one of the pathways through which they exert immunomodulatory and anti-inflammatory effects [38]. Our research identified a promotive effect of *IL12A* on SLE, aligning with the conclusions of other studies. Currently, there are three inhibitors of *IL12* (Ustekinumab, Ebdarokimab, and Briakinumab). Ustekinumab has been licensed to treat psoriasis and inflammatory bowel disease and has passed Phase II trials for SLE [39–41]. However, it failed a phase III trial in SLE (NCT03517722). We speculate that the failure may be attributed to targeting a single cytokine, which makes it difficult to address the complex immune dysregulation in SLE. Combining medications may enhance the potential of therapeutic efficacy. Ebdarokimab is under investigation to treat psoriasis. Briakinumab has been studied for a variety of autoimmune diseases, such as Crohn’s disease, psoriasis, and MS, but has not been studied for SLE yet. Considering the efficacy of targeted drugs and the risks associated with long-term use of corticosteroids, combining *IL12* inhibitors with corticosteroids holds promise for reducing steroid dosage and improving the potential for success with targeted therapies.

Heat Shock 70 KDa Protein 1A (*HSPA1A*) is a member of the Heat Shock Protein 70 (HSP70) family. Research indicates that HSP70 is associated with SLE [42]. Experiments conducted by Martina Mišunová’s team have shown that the expression of *HSPA1A* in SLE patients is greatly upregulated compared to healthy controls [43]. Our results indicate that *HSPA1A* is a risk factor for SLE. CD24FC is an inhibitor of *HSPA1A*/*HSPA1B*. In recent years, the immunoregulatory effects have been investigated for their efficacy against GVHD and severe acute respiratory syndrome. PheWAS indicated that high levels of *HSPA1A* may raise the risk of celiac disease and MS, yet act as a protective factor against RA and psoriasis. This indicates that drugs inhibiting this gene may benefit SLE patients but could lead to a higher risk of RA and psoriasis. Meanwhile, further exploration is needed to understand how *HSPA1A* mediates the pathogenesis of SLE.

The *NEU1* gene encodes sialidase-1, an enzyme that cleaves sialic acid. Cellular experiments discovered that in patients with active SLE, activated T cells exhibit reduced binding to Galectin-1 (Gal-1), and there is an increased gene expression rate of sialyltransferase and sialidase-1 (*NEU1*) [44]. Gal-1 is an immunomodulatory lectin that acts primarily to induce apoptosis in activated Th1 and Th17 T cell subsets [45]. The experiment also showed that the binding of Gal-1 was significantly increased after *NEU* treatment, suggesting that *NEU* treatment reduced T-cell resistance to GAL-1-mediated apoptosis. We found that an increase in *NEU1* expression, in addition to potentially reducing the risk of SLE, also lowers the risk of several other diseases, such as Type 1 diabetes, asthma, and diffuse diseases of connective tissue. Currently, most drugs targeting *NEU1* are inhibitors, so the development of drugs targeting *NEU1* requires further research.

H3 Clustered Histone 10 (*HIST1H3H*) encodes histone H3, which is essential for maintaining transcriptional regulation, DNA replication and repair, and chromosome stability. An increasing number of studies indicate that epigenetics plays an indispensable role in the immune regulation of SLE. For instance, studies have confirmed that the levels of trimethylation of histone 3 at lysine 27 (H3K27me3) are elevated in CD4 + T cells of SLE patients, indirectly promoting leukocyte adhesion and migration, thereby exacerbating SLE [46]. Interestingly, histone H3 in B cells exhibits low levels of acetylation, but how it affects antibody production remains unknown [47]. Our research has identified *HIST1H3H* as a risk factor for SLE, suggesting its impact on genetic associations. The expression of *HIST1H3H* leads to the production of histone H3, whose further modifications (acetylation or methylation) promote the activation or silencing of immune-related genes, ultimately resulting in immune dysregulation. SLE is the result of an imbalance in immune cell regulation, and *HIST1H3H*, a component of histone H3, is probably a critical factor affecting the immune balance in SLE. It is of great significance to develop new drugs for the treatment of SLE since there are no drugs targeting *HIST1H3H* currently.

We acknowledge limitations in this study. First, this research was confined to European populations, leading to ethnically specific conclusions that may not be validated across other racial groups. What’s more, lack of diversity in study cohorts may result in missing important genetic variants that are relevant to other populations, potentially affecting the efficacy of identified drug targets across races. Second, the derivation of core genes remained at the genomic level. Further exploration into transcriptional and translational mechanisms is necessary to fully elucidate these genes’ specific functions in SLE. This necessitates the validation of our conclusions by other researchers through multi-omics studies. Third, our results often rely on several key assumptions. One of the primary assumptions of MR is the assumption of no pleiotropy. Despite efforts to avoid pleiotropy using MR-Egger and sensitivity analyses, its existence cannot be completely ruled out. Pleiotropy may introduce bias in MR analyses, leading to false associations between gene expression and SLE risk. If there is genetic heterogeneity or multiple variations in certain regions associated with SLE, these colocalization signals could be weakened, leading to potentially flawed assumptions. Fourth, the sample size is not large enough for cases in the replication phase. This limitation affects the strength of validation: The smaller sample size reduces the robustness of the validation and is insufficient to detect the association of some rare variants with SLE, thus causing an increase in false negatives. Moreover, blood cis-eQTL data were used to investigate the effect of gene expression on SLE. However, SLE is a complex disease involving multiple organs, and the use of blood-derived eQTL alone may not capture tissue-specific genetic effects associated with other affected organs, such as the kidneys or skin. EQTL data from multiple organizations should be considered for inclusion in future studies. Another limitation of this paper is the non-response biases. Data sourced from the UK Biobank relied on participants’ self-reporting and medical records, which may introduce non-response bias if certain populations are less likely to accurately report their medical history. For example, individuals of lower socioeconomic status or from ethnic minority groups may be under-represented, leading to biased estimates of phenotypic associations.

## Conclusions

This study identified and validated five potentially druggable targets that hold promise for SLE. The genetic evidence presented supports the therapeutic potential of targeting these genes in SLE treatment. Clinical trials focusing on existing drugs and new medications that target these identified druggable genes may enhance the chances of successful treatments. Whatever, the mechanisms by which these candidate genes participate in SLE require further in-depth investigation.

### Supplementary Information

Below is the link to the electronic supplementary material.

Supplementary file1 (XLSX 11676 KB)

## Data Availability

All data is publicly available and can be obtained at the following site. Blood eOTL data were obtained from the eQTLGen Consortium (https://egtlgen.org/). SLE discovery data was from James Bentham’s study and downloaded on IEU OpenGWAS database (https://gwas.mrcieu.ac.uk/datasets/ebi-a-GCST003156/). SLE replication data was from FinnGen Release 10 (https://storage.cloud.google.com/finngen-public-data-r10/summary_stats/finngen_R10_SLE_FG.gz).

## Abbreviations

*SLE*: Systemic lupus erythematosus
*MR*: Mendelian randomization
*eQTL*: Expression quantitative trait loci
*GWAS*: Genome-wide association study
*PheWAS*: Phenome-wide association study
*RCT*: Randomized controlled trial
*IVs*: Instrumental variables
*FDR*: False discovery rate
*SNP*: Single nucleotide polymorphism
*IVW*: Inverse variance weighted
*OR*: Odds ratio
*CI*: Confidence interval
*RA*: Rheumatoid arthritis
*MS*: Multiple sclerosis
*GVHD*: Graft-versus-host disease.
